# COVID-19 Fog Symptoms Are Associated with Brain Metabolism and Platelet-to-Lymphocyte Ratio—A Cross-Sectional Analysis of the COVMENT Trial Baseline Data

**DOI:** 10.3390/jcm15051804

**Published:** 2026-02-27

**Authors:** Arkadiusz Lubas, Julia Bryłowska, Anna Grzywacz, Bartłomiej Włochacz, Agnieszka Giżewska, Mirosław Dziuk, Anna Klimkiewicz, Jakub Klimkiewicz

**Affiliations:** 1 Department of Internal Diseases, Nephrology and Dialysis, Military Institute of Medicine—National Research Institute, 04-141 Warsaw, Poland; 2 Faculty of Medicine, University of Warsaw, 02-089 Warsaw, Poland; 3 Department of Anesthesiology and Intensive Therapy, Military Institute of Medicine—National Research Institute, 04-141 Warsaw, Poland; 4 Nuclear Medicine Department, Military Institute of Medicine—National Research Institute, 04-141 Warsaw, Poland; 5 Department of Psychiatry, Medical University of Warsaw, 02-091 Warsaw, Poland; anna.klimkiewicz@wum.edu.pl

**Keywords:** cognitive dysfunction, COVID-19 brain fog, brain metabolism, neuroimmune alterations, COVMENT trial

## Abstract

**Background:** Post-COVID-19 cognitive impairment, commonly referred to as “brain fog,” represents a significant clinical problem, yet its underlying mechanisms remain incompletely understood. New research indicates that long-term cognitive consequences of SARS-CoV-2 infection may result from chronic immunological dysregulation and neurometabolic changes. Objective: We aimed to assess the associations between cognitive performance, cerebral glucose metabolism, and inflammatory markers in patients with COVID-19 brain fog symptoms. **Methods:** This study included 47 patients with post-COVID-19 cognitive complaints enrolled in the COVMENT trial. Cognitive performance was assessed using the Montreal Cognitive Assessment (MoCA). Brain glucose metabolism was evaluated with FDG PET-CT, and inflammatory markers, including C-reactive protein (CRP), monocyte-to-lymphocyte ratio, neutrophil-to-lymphocyte ratio, eosinophil-to-lymphocyte ratio, and platelet-to-lymphocyte ratio (PLR), were measured. Correlation analyses, logistic regression, and ROC analysis were performed to explore relationships between these factors. **Results:** A lower score of the MoCA abstraction domain correlated significantly with lower FDG uptake in multiple brain regions, including inferior parietal lobules and precuneus. Among inflammatory markers, only PLR demonstrated significant associations with both brain metabolism and abstraction performance. Lower PLR values were associated with greater neurometabolic impairment, and PLR < 130.1 was associated with abnormal abstraction performance. **Conclusions:** Post-COVID-19 cognitive dysfunction can be associated with selective neurometabolic alterations in brain regions supporting abstract reasoning. PLR seems to be associated with both cognitive performance and regional brain metabolism, suggesting a potential link between chronic immune dysregulation and neurocognitive impairment in post-COVID-19.

## 1. Introduction

COVID-19 is an infectious disease caused by the SARS-CoV-2 coronavirus from the Coronaviridae family. It presents with a variety of symptoms, including fever, cough, sore throat, loss of taste, smell, and diarrhea [1]. Emerging research by Zhao et al. indicates that COVID-19 is linked to a wide spectrum of neurological and cognitive symptoms that may persist long after the acute phase of infection, often described as long COVID syndrome [2]. Many patients struggle with the long-term consequences of COVID-19. The World Health Organization (WHO) defines “brain fog” as an informal name for a common complaint of impaired intellectual functioning among patients post-acute COVID-19. It is a catchall term for a range of cognitive problems, including disorientation, short-term memory loss, light-headedness, and difficulty focusing [3]. The Montreal Cognitive Assessment (MoCA) is a standardized neuropsychological test that assesses cognitive functions [4]. It contains several tasks that the patient must solve to assess various domains of cerebral function. Each MoCA domain adds a specific number of points to the total score, which is then combined to produce the final outcome. A total score of less than 26 is typically considered a sign of cognitive impairment, indicating deficiencies that less sensitive screening instruments may miss. This screening tool, therefore, enables individuals recovering from COVID-19 who report symptoms consistent with ‘brain fog’ to objectively assess whether measurable cognitive impairments are present [5,6].

COVID-19 is associated with profound disturbances of the immune system, particularly in severe and critical cases, where lymphopenia, lymphocyte activation, granulocyte and monocyte abnormalities, and elevated cytokine levels reflect a strong systemic inflammatory response [7]. According to the model discussed by Heneka’s group, systemic inflammation may disrupt the integrity of the blood–brain barrier (BBB), allowing inflammatory mediators to penetrate the brain parenchyma and induce chronic neuroinflammation [8]. Several inflammatory markers, such as the neutrophil-to-lymphocyte ratio (NLR), monocyte-to-lymphocyte ratio (MLR), platelet-to-lymphocyte ratio (PLR), and C-reactive protein (CRP), are widely used to assess immune activation and systemic inflammation in infectious and inflammatory diseases [9]. These markers typically increase during the acute phase of COVID-19. However, only limited data are available regarding their role in the long-term post-COVID period, because they may differ due to persistent immune dysregulation rather than severe or ongoing acute inflammation [10].

Recent neuroimaging work by Douaud et al. has demonstrated structural brain changes after COVID-19 [11]. Reduced grey matter thickness in the orbitofrontal cortex and parahippocampal gyrus regions, measured with Magnetic Resonance Imaging (MRI), implicated links with memory and executive functions. These alterations were associated with measurable cognitive decline. In addition, Toniolo et al. showed that COVID-19 infection may preferentially affect frontal brain regions, as evidenced by dysexecutive symptoms and reduced cerebral perfusion or metabolism. Frontal hypometabolism observed on 18F-FDG-PET, supported by evidence from various imaging modalities, including MRI, has been proposed as a potential neural substrate underlying executive and cognitive impairments reported in post-COVID patients [12]. Moreover, other neuroimaging studies, including those by Manganotti et al., have demonstrated brain changes in patients with persistent symptoms following COVID-19 [13]. Specifically, this study revealed significant hypometabolism in the precuneus and the inferior parietal lobe, regions considered crucial for abstract thinking processes. Even in the absence of obvious anatomical defects, such disruptions can impair brain metabolic function, as estimated by FDG ([18F] fluoro-2-deoxy-d-glucose) PET-CT, a recognized biomarker of neurodegeneration used to assess cerebral glucose metabolism and reflecting neuronal damage and synaptic dysfunction [14]. These data support the hypothesis that investigating the associations among immunological markers, brain metabolism, and COVID-related cognitive fog symptoms may offer a better understanding of its underlying pathogenesis.

This study aimed to evaluate the associations between COVID-19 fog symptoms, brain metabolic activity, and markers of inflammation in patients enrolled in the Randomized, Double-Blind, Placebo-Controlled Trial of the Efficacy and Safety of Tianeptine in the Treatment of COVID-19 Fog Symptoms in Patients After COVID-19 (COVMENT) funded by the Polish Medical Research Agency [15].

## 2. Materials and Methods

Patients who experienced COVID-19 brain fog and were eligible to participate in the COVMENT study by 14 January 2025 were included [12]. The patient’s written informed consent was required to take part in the clinical experiment. Inclusion criteria encompassed an age of at least 18 years and a positive SARS-CoV-2 test result using RT-PCR or a positive antigen test, indicating a history of COVID-19 infection. Subjective multidomain cognitive decline documented by patients following COVID-19 infection during screening and a MoCA score of less than 26 were prerequisites for the study. Exclusion criteria included hypersensitivity to fluorodeoxyglucose (FDG), history of drug or substance allergy, stroke, previous or planned brain surgery, organic central nervous system (CNS) damage, organic mental disorders, psychotic disorders, bipolar affective disorder, intellectual disability, or active depressive episodes requiring antidepressant treatment. Individuals with bipolar disorder in a first-degree relative were also excluded. As PET-CT testing requires the use of radiation, pregnancy and breastfeeding were exclusion criteria. Medical conditions such as uncontrolled diabetes, severe renal failure (eGFR < 30 mL/min/1.73 m^2^), and severe liver cirrhosis (Child-Pugh C) were grounds for exclusion, as were claustrophobia and chronic illnesses significantly worsening prognosis or quality of life. Patients were not eligible if they had an active or recent malignancy (within 5 years), except for radically treated basal cell carcinoma or cervical carcinoma in situ. Active viral, bacterial, fungal, tuberculous, or parasitic infections, as well as any other relevant diseases deemed by the Investigator to interfere with participation, also constituted exclusion criteria.

The COVMENT study (Military Institute of Medicine—National Research Institute; ABM/COVMENT/2021; EudraCT Number: 2022-000893-25, 10 November 2021) was approved by the Bioethics Committee of the Military Medical Chamber (No. 255/22; dated 27 May 2022) [Table S2]. This work concerns only patients who were eligible for the COVMENT study and were examined during the screening visit within the defined period.

### 2.1. Cognitive Assessment (MoCA)

During screening, the MoCA v.7.2 questionnaire in its Polish adaptation was completed [4]. The MoCA questionnaire includes tests assessing cognitive abilities in the form of visual-spatial and executive functions (clock drawing, drawing figure, and joining points—scored at a maximum of 5 points), naming skills (0–3 points), attention to numbers (0–2 points), letters (0–1 point), and subtraction (0–3 points), language functions in terms of repetition (0–2 points) and fluency (0–1 point), abstract reasoning functions (0–2 points), short-term memory and its selectivity (0–5 points), and allopsychic orientation (6 points). An additional 1 point is added for 12 or fewer years of education. The generally accepted threshold for diagnosing cognitive impairment is ≤26/30 points [16].

### 2.2. Blood Tests

On the first day of the screening visit, blood morphology with an automated differential blood count, serum C-reactive protein (CRP) [mg/dL], and ferritin [ng/mL] were assessed. Considered inflammatory markers in the form of eosinophil-to-lymphocyte ratio (ELR), MLR, NLR, and PLR were calculated by dividing the absolute count of eosinophils, monocytes, neutrophils, and platelets, respectively, by an absolute lymphocyte count.

### 2.3. FDG PET-CT

Brain glucose metabolism was assessed using 2-[^18^F] fluoro-2-deoxy-D-glucose (^18^F-FDG) positron emission tomography combined with computed tomography (FDG PET-CT). FDG, a glucose analogue, is taken up by metabolically active cells via glucose transporters and subsequently phosphorylated and retained intracellularly, enabling quantitative assessment of regional cerebral glucose metabolism [17]. FDG PET-CT enables evaluation of regional brain metabolism and is particularly useful for detecting functional abnormalities in the absence of overt structural changes. PET-CT examinations were performed after a minimum of 6 h of fasting and consuming 0.5–0.75 L of water one hour before the examination in patients whose blood glucose level at the screening visit was below 200 mg/dL. After intravenous administration of approximately 3 mL (150 MBq) of 18F-FDG solution, image acquisition began 30–40 min later. A GE OMNI LEGEND scanner (GE Healthcare, Milwaukee, WI, USA) was used for PET-CT imaging; images were acquired using a 384 × 384 matrix; scans took 10 min per bed. Images were assessed by two nuclear medicine physicians blinded to the clinical information. Images were reviewed and analyzed using Advantage Workstation (GE Healthcare) with dedicated clinical software (CortexID Suite ver. 2.1 ext. 6). The reference region selected for image intensity normalization was the pons. Regional glucose metabolic rates were estimated from the acquired PET-CT images, and the results were automatically compared with those of age-matched healthy controls. Study results were quantified as absolute uptake values and as deviations from reference values (Z-scores) using anatomically matched regions of interest. Comparison with a database of healthy individuals was performed using imaging software (GE CortexID Suite ver. 2.1 ext. 6). In the present study, FDG PET-CT was used to evaluate metabolic activity in brain regions involved in cognitive and abstract processing and to analyze its association with MoCA performance and inflammatory markers.

### 2.4. Statistical Analysis

The results are shown as means with standard deviation and the medians with interquartile range (IQR). For all variables, the Shapiro–Wilk test was used to assess normality. Depending on the distribution, Pearson’s or Spearman’s test was performed for correlation analysis, and the *t*-test or the U Mann–Whitney test for difference evaluation. The association with occurrence risk was tested with an univariable logistic regression analysis. Moreover, the ROC analysis using the Youden method was used to investigate the cut-off point. The two-tailed *p* < 0.05 was considered significant. All statistical tests were performed using Statistica v.13.3 software (StatSoft Inc., TIBCO Software Inc., Greenwood Village, CO, USA).

## 3. Results

**Figure 1:**
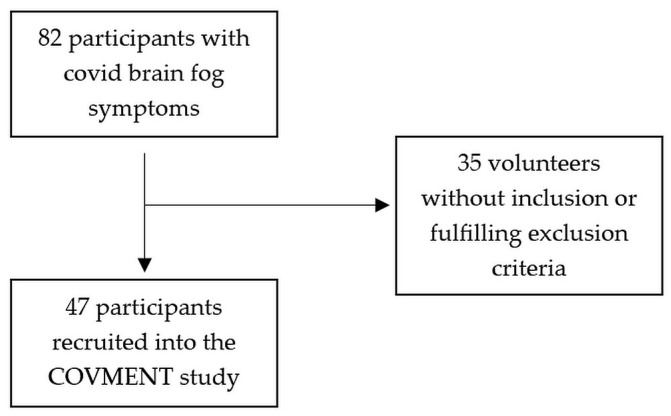
The study flow diagram.

Of the 82 patients who volunteered for the COVMENT trial until 14 January 2025, 47 (24 M, 23 F, age 50.7 ± 10.1) were ultimately recruited into the study (Figure 1). Results of the MoCA and considered inflammatory markers are presented in Table 1. There were no differences in the activity of contralateral brain regions estimated in FDG PET-CT (Table 2).

Although the total MoCA score was not correlated with the activity of any brain localization, the results of naming skills, attention—digits, repetition, fluency, and abstraction domain tests were associated with selected brain localizations (Table 3). The MoCA abstraction domain was most frequently correlated with brain FDG PET-CT localizations.

**Figure 2:**
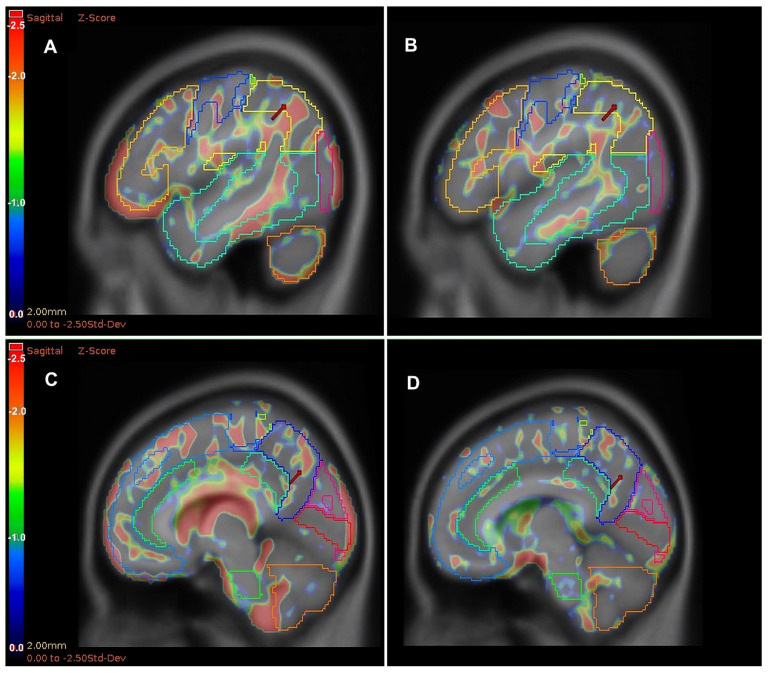
Visual presentation of hypometabolic brain areas in the FDG PET-CT scan in two patients with different PLRs. (**A**): Left parietal inferior lobulus delineated with yellow and marked with red arrow, uptake ratio = 0.93, Z-Score = −1.98. (**B**): Left parietal inferior lobulus delineated with yellow and marked with red arrow, uptake ratio = 1.74, Z-Score = 0.58. (**C**): Left precuneus delineated in navy blue and marked with red arrow, uptake ratio = 1.74, Z-Score = −0.59. (**D**): Left precuneus marked with red arrow, uptake ratio = 1.86, Z-Score = 0.40. For A and C, PLR = 98.3 and MoCA abstraction = 0/2; for B and D, PLR = 168.8 and MoCA abstraction = 2/2.

Among inflammatory markers, WBC significantly correlated only with activity in the right precuneus (r = −0.317; *p* = 0.030) and abstraction (r = −0.290; *p* = 0.048). Platelet count correlated with left and right temporal lateral brain localizations (r = 0.289; *p* = 0.049 and r = 0.333; *p* = 0.022, respectively), without association with MoCA domains. Although basophiles correlated significantly with 8 brain regions, they showed no significant relations with any MoCA domain tests. Conversely, PLR correlated significantly with metabolic activity in 5 brain regions and was substantially associated with abstraction (r = 0.325; *p* = 0.026). Other inflammatory markers were not correlated with MoCA or brain FDG PET-CT results. Among the symptoms of COVID-19 fog, the patients included most frequently reported short-term memory impairment (46/47), difficulty concentrating (44/47), balance impairment (34/47), chronic fatigue (26/47), headaches (22/47), shortened sleep (18/47), and depressed mood (15/47). However, these disturbances were not correlated with PLR, and only sleep disorders were substantially associated with cerebellar metabolic activity (r = −0.382; *p* = 0.008). Moreover, neither the metabolic activity of the examined brain areas nor the MoCA and PLR results correlated with the time since COVID-19. With the exception of the right and left primary visual and cerebellum regions, metabolic activity in all other brain regions negatively correlated with age (r = −0.591, *p* < 0.001 for brain mean uptake ratio). However, neither the PLR nor the MoCA total score nor the individual MoCA domain scores were related to age. After adjustment for age, the association of metabolic activity of left and right parietal inferior regions with PLR was slightly strengthened (r = 0.341; *p* = 0.020 and r = 0.379; *p* = 0.009, respectively), but the association with the MoCA abstraction domain was slightly weakened (r = 0.286; *p* = 0.054 and r = 0.303; *p* = 0.041, respectively). In an univariable logistic regression analysis, elevation of PLR was associated with a lower risk of improper abstraction domain test result (OR 0.983, 95% CI: 0.968–0.999; *p* = 0.040). The ROC analysis showed that PLR below the cut-off value of 130.1 can identify abnormal results in the abstraction domain test (sensitivity 60.0%, specificity 72.7%, AUC 0.673; *p* = 0.029). In addition, a comparative analysis showed a significant difference in metabolic activity of the right precuneus region between groups divided by PLR< or ≥130.1 (Table 4). Moreover, the differences detected in the left precuneus, right and left parietal inferior, right prefrontal lateral, and right temporal lateral regions, and in total brain metabolic activity were at the significance level (Table 4, Figure 2).

## 4. Discussion

In this cross-sectional observational study, we showed, for the first time in the literature, possible associations between brain glucose metabolism, cognitive dysfunction, and the platelet-to-lymphocyte ratio in patients with post-COVID-19 brain fog. The results of our research show that this disease can be associated with reduced brain metabolism in regions responsible for abstract reasoning: the parietal lobe and precuneus, as assessed by FDG PET-CT, which correlate with lower scores of the MoCA abstraction domain.

Analysis of the MoCA abstraction domain revealed positive correlations with metabolic activity in multiple brain regions, including the cerebellum, bilateral occipital lateral cortex, and bilateral inferior and superior parietal lobes (Table 3). Among these regions, the strongest correlations were observed in the inferior parietal lobules, with r = 0.317 for the left hemisphere and r = 0.310 for the right hemisphere, consistent with their known involvement in abstract reasoning. This is consistent with the study by Xu et al., which found that explicit logical thinking, such as transitive inference, depends on the inferior parietal cortex [18]. Xu and coworkers conducted an experimental functional MRI (fMRI) study in healthy adults using a transitive inference task to examine the neural basis of abstract logical reasoning. The controlled task-based design allowed the authors to demonstrate a specific involvement of the inferior parietal cortex in explicit inference processes. In our study, correlation analysis indicated that lower performance on the MoCA abstraction tasks was associated with reduced regional cerebral glucose metabolism in areas supporting abstract cognitive processing. Moreover, consistent with our findings, a recent study by Manganotti et al. using the same neuroimaging modality and investigating patients with persistent post-COVID-19 symptoms, demonstrated significant cerebral hypometabolism [13]. In this study, FDG PET analysis revealed hypometabolism distributed across eight distinct metabolic clusters. Importantly, although hypometabolic changes were observed in multiple brain regions, including temporal and other cortical areas, significant clusters were also identified within both the left and right parietal lobes. This parietal involvement closely parallels our observations, supporting the notion that parietal hypometabolism is a reproducible finding in post-COVID-19 cognitive impairment as assessed by FDG PET imaging.

The mean MoCA abstraction score observed in our cohort was 1.38, which is below the maximum score of 2 points expected in cognitively healthy individuals. This finding indicates a measurable impairment in abstract reasoning among patients with post-COVID-19 cognitive complaints. Importantly, our results are consistent with a previous report by Sirait et al., demonstrating reduced abstraction scores in patients with post-COVID-19 [19]. These researchers conducted a cross-sectional observational study among healthcare workers who had recovered from COVID-19, assessing cognitive function with the Montreal Cognitive Assessment (MoCA) and quality-of-life measures. The study population represented a post-COVID condition cohort, allowing the authors to identify domain-specific cognitive impairments. This study showed that the language and abstraction domains had significantly lower mean scores of 1.88 ± 0.69 and 1.43 ± 0.64, respectively, suggesting that these domains may be more vulnerable to COVID-19-induced consequences [19]. These results support our findings that post-COVID-19 brain fog may be associated with impaired abstract reasoning as captured by the MoCA abstraction domain. Similar observations were reported in the Brutto et al. study, which conducted MoCA tests in 78 participants before COVID-19 and 6 months after, and found a notable decline in MoCA scores among COVID-19 survivors [20]. However, this study only reports on the total MoCA score, without analyzing individual domains. In contrast, our analysis focused on domain-specific cognitive performance, enabling us to assess impairments within each domain and identify selective deficits in abstract reasoning. Despite these methodological differences, both studies consistently demonstrate an association between long COVID and impaired cognitive performance as assessed by the MoCA, supporting the presence of post-COVID-19 cognitive dysfunction.

Our findings suggest that the platelet-to-lymphocyte ratio may serve as a relevant marker of persistent neurocognitive involvement in prolonged COVID-19 rather than a simple indicator of acute systemic inflammation. The association between inflammatory biomarkers and neurocognitive outcomes is further supported by studies in acute COVID-19, such as that by Gutowski et al. and Di Giorgio et al., which demonstrated that elevated markers of systemic inflammation are associated with an increased risk of delirium, which is a predictor of persistent cognitive dysfunction [21,22]. However, it is crucial to note that the inflammatory patterns in acute and chronic phases are not directly comparable. While delirium in acute COVID-19 reflects a state of pronounced systemic inflammation, our findings in prolonged post-COVID-19 suggest that reduced PLR, in the presence of normal CRP values, may instead reflect chronic immune dysregulation rather than ongoing acute inflammation. Together, these observations indicate that distinct inflammatory patterns may underlie acute and chronic neurocognitive manifestations of COVID-19. While elevated PLR and NLR are well documented in severe and acute COVID-19 as reflections of inflammatory activation and immune dysregulation, the behaviour of PLR in the long-term post-COVID-19 setting appears to differ [23]. In our cohort, PLR was associated with cerebral metabolic activity across five brain regions: the left and right parietal inferior regions, the right precuneus, and the left and right temporal lateral regions (Table 3). Notably, lower PLR values were associated with abnormal abstraction test results in MoCA and with reduced glucose metabolism in key regions implicated in higher-order cognitive processing, including the precuneus and the inferior parietal cortex. Although moderate, PLR’s ability to discriminate abnormal abstraction performance, as demonstrated by ROC analysis, further supports its potential relevance as a marker of post-COVID-19 cognitive dysfunction. Importantly, stratification of patients by the PLR cut-off of 130.1 revealed that individuals with PLR ≥ 130.1 exhibited significantly higher cerebral glucose metabolism than those with PLR < 130.1. This finding suggests that lower PLR values can be associated with more pronounced neurometabolic impairment, whereas higher PLR may reflect a relatively preserved metabolic state in brain regions supporting abstract cognitive processing. In a large population-based prospective cohort study by Fest et al., including individuals aged 45 years and older, reference values for inflammatory markers were established using absolute blood counts. The authors reported a mean platelet-to-lymphocyte ratio (PLR) of approximately 120 in the general population [24]. This value is closely aligned with the PLR cut-off of 130.1 reported in our study, supporting its clinical relevance. Although most scientific studies on active neurodegenerative and neuroinflammatory diseases identify elevated PLR as a marker of worse prognosis, lower PLR values had a significant predictive value for mortality in the course of hemorrhagic stroke (which is probably associated with thrombocytopenia) [25,26,27,28]. On the other hand, a greater prognostic significance of reduced PLR was observed in severe viral infections, e.g., a significantly higher risk of developing hemorrhagic vs. non-hemorrhagic dengue fever [29]. In a study of 204 children with infective mononucleosis, 109 pediatric patients with other viral infections, and 86 healthy children, Wei et al. found that decreased PLR during the disease period was a significant prognostic marker for recognizing infectious mononucleosis [30]. These studies seem to confirm the association between reduced PLR and immunological alterations in the course of severe viral diseases. To date, no previous studies have directly examined the relationship between platelet-to-lymphocyte ratio and brain glucose metabolism measured by FDG PET-CT in the context of long COVID-19, making our findings a novel contribution to the field.

Considering the association between PLR value and MoCA scores, similar results were observed in the study conducted by Nolasco-Rosales et al., where low PLR (<103.9) was associated with impaired delayed recall in the MoCA (*p* = 0.040), suggesting that reduced PLR may reflect persistent neurocognitive involvement in post-COVID-19 conditions [31]. In this study, a descriptive cross-sectional design was applied to a cohort of 51 Mexican healthcare workers with post-COVID-19 condition, with a median post-infection duration of 14 months. The authors evaluated cognitive performance using both the MoCA and MMSE tests and examined inflammatory markers, including PLR, NLR, and MLR. Unlike our investigation, which also incorporated neurometabolic measures via FDG PET-CT, this study focused on peripheral inflammatory markers and global cognitive testing without concurrent neuroimaging, highlighting complementary evidence for a link between PLR and specific cognitive deficits in post-COVID-19 populations. On the other hand, Nolasco-Rosales et al. examined post-COVID-19 patients and used the same type of cognitive test and inflammatory marker as in our study. Together, these findings suggest that, in prolonged COVID-19, alterations in PLR may reflect chronic immune dysregulation and neuroimmune interactions that are associated with neurometabolic changes underlying cognitive impairment, rather than ongoing acute inflammation.

Although we found promising results, our study has several limitations that should be acknowledged. First, the relatively small sample size limits the analyses’ statistical power and may reduce the generalizability of the findings. A larger study population could provide more statistically significant results. Second, the lack of a healthy control group without post-COVID-19 cognitive symptoms limits direct comparisons of cerebral metabolism, PLR changes, and cognitive performance between individuals with long COVID-19 and healthy controls. Inclusion of a control group undergoing blood and MoCA testing with FDG PET-CT would allow for a more precise evaluation of COVID-19-related neurometabolic and cognitive changes. Additionally, the limited sample size and effect sizes of the investigated relationships do not allow for false discovery rate analysis, which is helpful for multiple testing. Furthermore, ROC analysis shows modest discrimination and should, therefore, be interpreted with caution. Moreover, cognitive assessment, neuroimaging, and inflammatory markers (MoCA, FDG PET-CT, and PLR) were obtained at a single time point. Consequently, the longitudinal course of these abnormalities remains unclear, whether these alterations in PLR precede cognitive impairment and neurometabolic changes, or whether these findings are transient or permanent. Reassessing the same cohort at a longer follow-up interval, as is planned in the COVMENT study, would provide valuable insight into the temporal dynamics of these findings. Finally, although MoCA is widely used as a screening tool, it may not fully capture the complexity and domain-specific nature of cognitive deficits observed in long COVID-19. Future studies would benefit from more detailed neuropsychological assessments to better characterize cognitive impairment in this population.

## 5. Conclusions

Patients with post-COVID-19 cognitive symptoms, referred to as COVID-19 brain fog, exhibit selective impairment in abstract reasoning, which may be associated with reduced cerebral glucose metabolism in the inferior parietal lobes and precuneus, as assessed by FDG PET-CT. These findings are consistent with the concept that post-COVID-19 brain fog may involve predominant neurometabolic alterations. The platelet-to-lymphocyte ratio seems to be associated with both cognitive performance and regional brain metabolism, suggesting a potential link between chronic immune dysregulation and neurocognitive impairment in post-COVID-19. Although promising, our findings indicate only PLR associations and do not establish a causal relationship, which requires confirmation in longitudinal studies that combine cognitive testing, neuroimaging, and immunological profiling.

## Figures and Tables

**Table 1:** Results of the MoCA test and inflammation markers in recruited patients.

Test/Function	Mean	SD	Median	IQR
MoCA Visuospatial function *	2.936	1.030	3	2
MoCA Naming skills	2.957	0.204	3	0
MoCA Attention—digits	1.638	0.486	2	1
MoCA Attention—letters	0.766	0.43	1	0
MoCA Attention—substraction	2.511	0.804	3	1
MoCA Repetition	1.660	0.563	2	1
MoCA Fluency	0.319	0.471	0	1
MoCA Abstraction	1.383	0.645	1	1
MoCA Short-Term Memory	3.149	1.335	3	2
MoCA Allopsychic Orientation	5.787	0.463	6	0
MoCA Total Score	23.128	1.740	24	2
WBC (1 × 10^9^/L)	6.744	1.408	6.770	1.900
Basophiles (1 × 10^9^/L)	0.050	0.024	0.050	0.030
Eosinophils (1 × 10^9^/L)	0.183	0.158	0.150	0.160
Lymphocytes (1 × 10^9^/L)	1.940	0.441	1.920	0.680
Monocytes (1 × 10^9^/L)	0.540	0.130	0.520	0.150
Neutrophils (1 × 10^9^/L)	4.011	1.129	4.130	1.650
Platelets (1 × 10^9^/L)	257.681	66.702	248.000	102.000
ELR (ratio)	0.094	0.074	0.083	0.065
MLR (ratio)	0.289	0.082	0.269	0.084
NLR (ratio)	2.139	0.709	1.996	0.996
PLR (ratio)	138.637	42.901	134.066	65.030
Ferritine (ng/mL)	117.638	100.218	85.000	143.000
CRP (mg/dL)	0.196	0.238	0.100	0.110

CRP—C-reactive protein; ELR—eosinophil-to-lymphocyte ratio; MoCA—Montreal Cognitive Assessment; MLR—monocyte-to-lymphocyte ratio; NLR—neutrophil-to-lymphocyte ratio; PLR—platelet-to-lymphocyte ratio; WBC—white blood count; * visuospatial function—sum of two test results: clock drawing, and drawing figure and joining points.

**Table 2:** Comparison of metabolic activity (FDG PET-CT) of left and right cerebral regions.

Cerebral Region	Uptake Ratio Results		Z-Score		Significance—*p*
	Mean [Median]	SD [IQR]	Mean [Median]	SD [IQR]	Results L: R
anterior cingulate L	1.425	0.139	0.031	0.902	0.711
anterior cingulate R	[1.400]	[0.190]	[−0.260]	[1.900]	
cerebellum whole	1.259	0.064	−0.329	0.901	-
occipital lateral L	1.810	0.163	1.326	1.379	0.834
occipital lateral R	1.817	0.162	1.349	1.342	
parietal inferior L	1.635	0.154	[−0.220]	[1.480]	0.372
parietal inferior R	1.607	0.143	−0.221	1.096	
parietal superiol L	1.562	0.161	[0.170]	[1.410]	0.808
parietal superiol R	1.571	0.177	−0.095	1.243	
posterior cingulate L	1.840	0.169	0.297	0.954	0.913
posterior cingulate R	1.844	0.170	0.257	1.023	
precuneus L	1.741	0.147	−0.215	0.962	0.961
precuneus R	[1.710]	[0.190]	−0.213	1.004	
prefrontal lateral L	1.721	0.178	0.287	1.311	0.561
prefrontal lateral R	1.700	0.157	[−0.030]	[1.900]	
prefrontal medial L	1.559	0.142	[−0.220]	[1.050]	0.425
prefrontal medial R	1.535	0.141	−0.192	1.069	
primary visual L	2.178	0.278	2.159	1.694	0.606
primary visual R	2.149	0.268	2.221	1.740	
sensorimotor L	1.644	0.148	0.306	1.064	0.100
sensorimotor R	1.597	0.129	−0.021	0.963	
temporal lateral L	1.457	0.102	−0.056	0.866	0.288
temporal lateral R	1.433	0.110	−0.303	0.983	
temporal mesial L	1.102	0.062	−0.201	0.894	0.456
temporal mesial R	1.092	0.070	−0.281	0.990	

L—left, R—right.

**Table 3:** Correlation coefficients of significant associations between MoCA test results, metabolic activity of brain regions, and inflammatory markers.

	MoCA Naming Skills	MoCA Attention—Digits	MoCA Repetition	MoCA Fluency	MoCA Abstraction	Basophils	PLR
cerebellum whole					0.304		
occipital lateral L		−0.294			0.294	−0.332	
occipital lateral R					0.298	−0.330	
parietal inferior L			−0.318		0.317		0.302
parietal inferior R					0.310		0.324
parietal superiol L					0.309	−0.298	
parietal superiol R					0.300	−0.325	
posterior cingulate L			−0.294				
posterior cingulate R			−0.326		0.290		
precuneus L			−0.291			−0.290	
precuneus R						−0.300	0.330
prefrontal lateral L	−0.299					−0.320	
prefrontal medial R			−0.319	0.322			
sensorimotor R						−0.416	
temporal lateral L	−0.300		−0.296				0.289
temporal lateral R	−0.296						0.333

MoCA—Montreal Cognitive Assessment; L—left; PLR—platelet-to-lymphocyte ratio; R—right.

**Table 4:** Comparison of brain FDG PET-CT uptake ratio results across groups divided by PLR cut-off value.

Variable	PLR < 130.1 n = 20		PLR ≥ 130.1 n = 27		Significance—*p*
	Mean [Median]	SD [IQR]	Mean [Median]	SD [IQR]	
anterior cingulate L	1.409	0.111	1.437	0.158	0.495
anterior cingulate R	1.398	0.127	1.439	0.162	0.359
cerebellum whole	1.257	0.066	1.260	0.064	0.892
occipital lateral L	1.767	0.151	1.843	0.166	0.112
occipital lateral R	1.773	0.151	1.850	0.165	0.106
parietal inferior L	1.590	0.148	1.669	0.152	0.081
parietal inferior R	1.563	0.136	1.640	0.141	0.066
parietal superior L	1.522	0.143	1.592	0.169	0.140
parietal superior R	1.538	0.159	1.596	0.187	0.270
posterior cingulate L	1.796	0.147	1.873	0.179	0.119
posterior cingulate R	1.798	0.146	1.878	0.180	0.110
precuneus L	1.699	0.119	1.772	0.159	0.090
precuneus R	[1.690]	[0.135]	1.789	0.154	0.039
prefrontal lateral L	1.672	0.134	1.757	0.200	0.109
prefrontal lateral R	1.652	0.129	1.736	0.168	0.068
prefrontal medial L	1.527	0.114	1.583	0.157	0.183
prefrontal medial R	1.498	0.127	1.563	0.147	0.126
primary visual L	2.144	0.289	2.204	0.272	0.466
primary visual R	2.101	0.268	2.185	0.267	0.288
sensorimotor L	1.605	0.140	1.674	0.148	0.115
sensorimotor R	1.566	0.130	1.620	0.125	0.151
temporal lateral L	1.430	0.085	1.477	0.110	0.123
temporal lateral R	1.400	0.107	1.458	0.107	0.072
temporal mesial L	1.095	0.061	1.108	0.063	0.473
temporal mesial R	1.084	0.075	1.098	0.067	0.485
total brain activity	40.579	2.644	42.100	3.235	0.092

L—left, PLR—platelet-to-lymphocyte ratio; R—right.

## Data Availability

The data presented in this study are available on request from the corresponding author.

## Supplementary Materials

The following supporting information can be downloaded at https://www.mdpi.com/article/10.3390/jcm15051804/s1. Table S1: COVMENT Researchers; Table S2: CONSORT 2025 checklist of information to include when reporting a randomized trial * [32].

## References

[1] Velavan T.P., Meyer C.G. (2020). The COVID-19 Epidemic. Trop. Med. Int. Health.

[2] Zhao S., Toniolo S., Hampshire A., Husain M. (2023). Effects of COVID-19 on Cognition and Brain Health. Trends Cogn. Sci..

[3] Nouraeinejad A. (2023). Brain Fog as a Long-Term Sequela of COVID-19. SN Compr. Clin. Med..

[4] Gierus J., Mosiołek A., Koweszko T., Kozyra O., Wnukiewicz P., Łoza B., Szulc A. (2015). Montrealska Skala Oceny Funkcji Poznawczych MoCA 7.2—Polska Adaptacja Metody i Badania Nad Równowaznosci. Psychiatr. Pol..

[5] Masserini F., Pomati S., Cucumo V., Nicotra A., Maestri G., Cerioli M., Giacovelli L., Scarpa C., Larini L., Cirnigliaro G. (2024). Assessment of Cognitive and Psychiatric Disturbances in People with Post-COVID-19 Condition: A Cross-Sectional Observational Study. CNS Spectr..

[6] Klimkiewicz J., Pankowski D., Wytrychiewicz-Pankowska K., Klimkiewicz A., Siwik P., Klimczuk J., Lubas A. (2022). Analysis of the Relationship among Cognitive Impairment, Nutritional Indexes and the Clinical Course among COVID-19 Patients Discharged from Hospital—Preliminary Report. Nutrients.

[7] Yang L., Liu S., Liu J., Zhang Z., Wan X., Huang B., Chen Y., Zhang Y. (2020). COVID-19: Immunopathogenesis and Immunotherapeutics. Signal. Transduct. Target. Ther..

[8] Heneka M.T., Carson M.J., Khoury J.E., Landreth G.E., Brosseron F., Feinstein D.L., Jacobs A.H., Wyss-Coray T., Vitorica J., Ransohoff R.M. (2015). Neuroinflammation in Alzheimer’s Disease. Lancet Neurol..

[9] Damar Çakırca T., Torun A., Çakırca G., Portakal R.D. (2021). Role of NLR, PLR, ELR and CLR in Differentiating COVID-19 Patients with and without Pneumonia. Int. J. Clin. Pract..

[10] Zotova N., Zhuravleva Y., Chereshnev V., Gusev E. (2023). Acute and Chronic Systemic Inflammation: Features and Differences in the Pathogenesis, and Integral Criteria for Verification and Differentiation. Int. J. Mol. Sci..

[11] Douaud G., Lee S., Alfaro-Almagro F., Arthofer C., Wang C., Lange F., Andersson J., Griffanti L., Duff E., Jbabdi S. (2021). Brain Imaging before and after COVID-19 in UK Biobank. medRxiv.

[12] Toniolo S., Di Lorenzo F., Scarioni M., Frederiksen K.S., Nobili F. (2021). Is the Frontal Lobe the Primary Target of SARS-CoV-2?. J. Alzheimers Dis..

[13] Manganotti P., Iscra K., Furlanis G., Michelutti M., Miladinović A., Menichelli A., Cerio I., Accardo A., Dore F., Ajčević M. (2025). Mapping Brain Changes in Post-COVID-19 Cognitive Decline via FDG PET Hypometabolism and EEG Slowing. Sci. Rep..

[14] Carneiro C.d.G., Faria D.d.P., Coutinho A.M., Ono C.R., Duran F.L.d.S., da Costa N.A., Garcez A.T., da Silveira P.S., Forlenza O.V., Brucki S.M.D. (2022). Evaluation of 10-Minute Post-Injection11C-PiB PET and Its Correlation With18F-FDG PET in Older Adults Who Are Cognitively Healthy, Mildly Impaired, or with Probable Alzheimer’s Disease. Braz. J. Psychiatry.

[15] Anna Klimkiewicz Randomized, Double-Blind, Placebo-Controlled Trial of the Efficacy and Safety of Tianeptine in the Treatment of Covid Fog Symptoms in Patients After COVID-19 (COVMENT), NCT06012552, ClinicalTrials.Gov. NCT06012552.

[16] Nasreddine Z.S., Phillips N.A., Bédirian V., Charbonneau S., Whitehead V., Collin I., Cummings J.L., Chertkow H. (2005). The Montreal Cognitive Assessment, MoCA: A Brief Screening Tool for Mild Cognitive Impairment. J. Am. Geriatr. Soc..

[17] Basu S., Hess S., Nielsen Braad P.E., Olsen B.B., Inglev S., Høilund-Carlsen P.F. (2014). The Basic Principles of FDG-PET/CT Imaging. PET Clin..

[18] Xu B., Wu J., Xiao H., Münte T.F., Ye Z. (2024). Inferior Parietal Cortex Represents Relational Structures for Explicit Transitive Inference. Cerebral Cortex.

[19] Sirait S.R.A., Sinaga B.Y.M., Tarigan A.P., Wahyuni A.S. (2024). Factors Associated with Cognitive Impairment and the Quality-of-Life among COVID-19 Survivors Working as Healthcare Workers. Narra J..

[20] Del Brutto O.H., Rumbea D.A., Recalde B.Y., Mera R.M. (2022). Cognitive Sequelae of Long COVID May Not Be Permanent: A Prospective Study. Eur. J. Neurol..

[21] Gutowski M., Klimkiewicz J., Michałowski A., Ordak M., Możański M., Lubas A. (2023). ICU Delirium Is Associated with Cardiovascular Burden and Higher Mortality in Patients with Severe COVID-19 Pneumonia. J. Clin. Med..

[22] Di Giorgio A., Mirijello A., De Gennaro C., Fontana A., Alboini P.E., Florio L., Inchingolo V., Zarrelli M., Miscio G., Raggi P. (2022). Factors Associated with Delirium in COVID-19 Patients and Their Outcome: A Single-Center Cohort Study. Diagnostics.

[23] Khalid A.M.A.M., Suliman A.M., Abdallah E.I., Abakar M.A.A., Elbasheir M.M., Muddathir A.M., Aldakheel F.M., Shaya A.S.B., Alfahed A., Alharthi N.S. (2022). Influence of COVID-19 on Lymphocyte and Platelet Parameters among Patients Admitted Intensive Care Unit and Emergency. Eur. Rev. Med. Pharmacol. Sci..

[24] Fest J., Ruiter R., Ikram M.A., Voortman T., Van Eijck C.H.J., Stricker B.H. (2018). Reference Values for White Blood-Cell-Based Inflammatory Markers in the Rotterdam Study: A Population-Based Prospective Cohort Study. Sci. Rep..

[25] Madetko N., Migda B., Alster P., Turski P., Koziorowski D., Friedman A. (2022). Platelet-to-Lymphocyte Ratio and Neutrophil-to-Lymphocyte Ratio May Reflect Differences in PD and MSA-P Neuroinflammation Patterns. Neurol. Neurochir. Pol..

[26] Liu G., Zhou Y., Ding H., Chen L., Chen L., Yang S. (2025). Relationship between the Platelet-to-Lymphocyte Ratio and in-Hospital Mortality of Ischemic Stroke Patients in the Intensive Care Unit. Front. Aging Neurosci..

[27] Carnero Contentti E., López P.A., Criniti J., Pettinicchi J.P., Cristiano E., Patrucco L., Lazaro L., Alonso R., Fernández Liguori N., Tkachuk V. (2022). Platelet-to-Lymphocyte Ratio Differs between MS and NMOSD at Disease Onset and Predict Disability. Mult. Scler. Relat. Disord..

[28] Rzepiński Ł. (2022). ‘Primary Progressive Multiple Sclerosis Overlapping with Anti-GAD and Anti-Hu Antibodies Positive Neurological Syndromes’—Clinical Considerations. Neurol. Neurochir. Pol..

[29] Böer L.M., Junqueira I.C., Nascimento T.C.D., Guilarde A.O., Féres V.C.d.R., de Alcântara K.C. (2024). Monocyte-lymphocyte, neutrophil-lymphocyte, and platelet-lymphocyte ratios as inflammatory biomarkers of clinical dengue severity. Biosci. J..

[30] Wei Y., Zhang K., Wang P., Yuan E. (2025). “Infectious Mononucleosis” Flag, High-Fluorescence Lymphocyte Percentage, and Platelet-to-Lymphocyte Ratio as Diagnostic and Prognostic Biomarkers for Infectious Mononucleosis in Chinese Children. Ital. J. Pediatr..

[31] Nolasco-Rosales G.A., Alonso-García C.Y., Hernández-Martínez D.G., Villar-Soto M., Martínez-Magaña J., Genis-Mendoza A.D., González-Castro T.B., Tovilla-Zarate C.A., Guzmán-Priego C.G., Martínez-López M.C. (2023). Aftereffects in Epigenetic Age Related to Cognitive Decline and Inflammatory Markers in Healthcare Personnel with Post-COVID-19: A Cross-Sectional Study. Int. J. Gen. Med..

[32] Hopewell S., Chan A.W., Collins G.S., Hróbjartsson A., Moher D., Schulz K.F., Tunn R., Aggarwal R., Berkwits M., Berlin J.A. (2025). CONSORT 2025 Statement: Updated guideline for reporting randomised trials. BMJ.

